# The R2R3-MYB transcription factor PaMYB10 is involved in anthocyanin biosynthesis in apricots and determines red blushed skin

**DOI:** 10.1186/s12870-019-1898-4

**Published:** 2019-07-01

**Authors:** Wanpeng Xi, Jing Feng, Yu Liu, Shikui Zhang, Guohua Zhao

**Affiliations:** 1 College of Food Science, Southwest University, Chongqing, 400715 China; 2 College of Horticulture and Landscape Architecture, Southwest University, Chongqing, 400716 China; 3 Agriculture National Fruit Tree Germplasm Repository, Xinjiang Academy of Agricultural Sciences, Luntai, Xinjiang, 841600 China

**Keywords:** *Prunus armeniaca*, Anthocyanin, MYB transcription factor, WGCNA, Cloning, qRT-PCR

## Abstract

**Background:**

The majority of apricot (*Prunus armeniaca L.*) cultivars display orange or yellow background skin, whereas some cultivars are particularly preferred by consumers because of their red blushed skin on the background.

**Results:**

In this study, two blushed (‘Jianali’ and ‘Hongyu’) and two nonblushed (‘Baixing’ and ‘Luntaixiaobaixing’) cultivars were used to investigate the formation mechanism of blushed skin in apricots. High-performance liquid chromatography (HPLC) analysis showed that the blushed cultivars accumulated higher cyanidin-3-O-glucoside, cyanidin-3-O-rutinoside and peonidin-3-O-rutinoside levels during fruit ripening than the nonblushed cultivars. Based on coexpression network analysis (WGCNA), a putative anthocyanin-related R2R3-MYB, *PaMYB10*, and seven structural genes were identified from transcriptome data. The phylogenetic analysis indicated that *PaMYB10* clustered in the anthocyanin-related MYB clade. Sequence alignments revealed that PaMYB10 contained a bHLH-interaction motif ([DE]Lx2[RK]x3Lx6Lx3R) and an ANDV motif. Subcellular localization analysis showed that PaMYB10 was a nuclear protein. Real-time qRT-PCR analysis demonstrated that the transcript levels of *PaMYB10* and seven genes responsible for anthocyanin synthesis were significantly higher in blushed than in nonblushed apricots, which was consistent with the accumulation of anthocyanin. In addition, bagging significantly inhibited the transcript levels of *PaMYB10* and the structural genes in ‘Jianali’ and blocked the red coloration and anthocyanin accumulation. Transient *PaMYB10* overexpression in ‘Luntaixiaobaixing’ fruits resulted in the red blushed skin at the maturation stage.

**Conclusions:**

Taken together, these data reveal that three anthocyanins are responsible for the blushed skin of apricots, identify *PaMYB10* as a positive regulator of anthocyanin biosynthesis in apricots, and demonstrate that blush formation depends on light.

**Electronic supplementary material:**

The online version of this article (10.1186/s12870-019-1898-4) contains supplementary material, which is available to authorized users.

## Background

Apricot (*Prunus armeniaca* L.) is a widely cultivated temperate fruit tree species [1] with fruit that contains many types of carotenoids and presents with orange or yellow skin. Xinjiang is one of the primary centers of apricot domestication worldwide and is also the main cultivation area in China; almost 200 varieties are cultivated in this region [2]. Among these varieties, some cultivars with a red blush on the orange or yellow background skin are particularly preferred by consumers due to their beautiful color and high nutritional value [3, 4]. However, the mechanism underlying blush formation in these cultivars is unknown.

Anthocyanins are water-soluble flavonoid pigments that accumulate in the vacuoles of many flowers, fruits, seeds, and vegetables, where they contribute to the red, purple and blue coloration [5]. As the primary color determinant and antioxidant components, the accumulation of anthocyanin pigments in fruit is an important indicator of health-promoting property [6, 7]. Therefore, understanding the regulation of anthocyanin biosynthesis in fruits is important for development of anthocyanin-rich foods for our diet.

Anthocyanins are synthesized from a branch of the flavonoid pathway, which has been extensively studied in many plants [8–10]. In general, anthocyanin biosynthesis at the transcriptional level is controlled by a complex of DNA-binding R2R3 MYB transcription factors, MYC-like basic helix–loop–helix (bHLH) proteins and WD40 proteins [11–13]. MYB members of this complex can separately play the regulatory role in anthocyanin biosynthesis [14, 15], and have been identified in various fruit crops, including grapes [16], apples [17], pears [18] and peaches [19].

MYB TFs have been reported to have diverse functions in controlling plant pathways, such as secondary metabolism, development, signal transduction, and disease resistance [20]. These TFs are characterized by a structurally conserved DNA-binding domain consisting of single or multiple imperfect repeats. Among these MYB TFs, the two repeat class (R2R3) is the largest, with 133 R2R3 MYB TFs reported in *Arabidopsis* [21, 22] and 108 reported in grapes [23]. It has been clearly elucidated that R2R3 MYB TFs are the key to determine the spatial and temporal patterning of anthocyanin production in most plant species [7, 15, 24]. Hence, the isolation and characterization of MYB TFs associated with anthocyanin biosynthesis is an important step towards understanding and manipulating fruit coloration [25].

Recently, great progress has been made in elucidating the molecular control of the anthocyanin pathway in many horticulture crops [14]. Anthocyanin biosynthesis is enhanced by sunlight in apples, pears, and peaches [26]. In apricots, although blushed skin is observed in some cultivars, no information about the mechanism of its development is available at present. In the present study, we determined the main pigments contributing to fruit blushed skin and identified one R2R3 MYB transcription factor and seven structural genes of the anthocyanin biosynthetic pathway based on coexpression network analysis (WGCNA). The potential roles of MYB in anthocyanin accumulation in apricots were investigated by bagging and transient overexpression. Our results suggest that *PaMYB10* is responsible for controlling anthocyanin biosynthesis and is involved in the development of blushed skin in apricots and that the process is regulated by light.

## Results

### Changes in basic quality parameters during fruit development and ripening

**Fig. 1:**
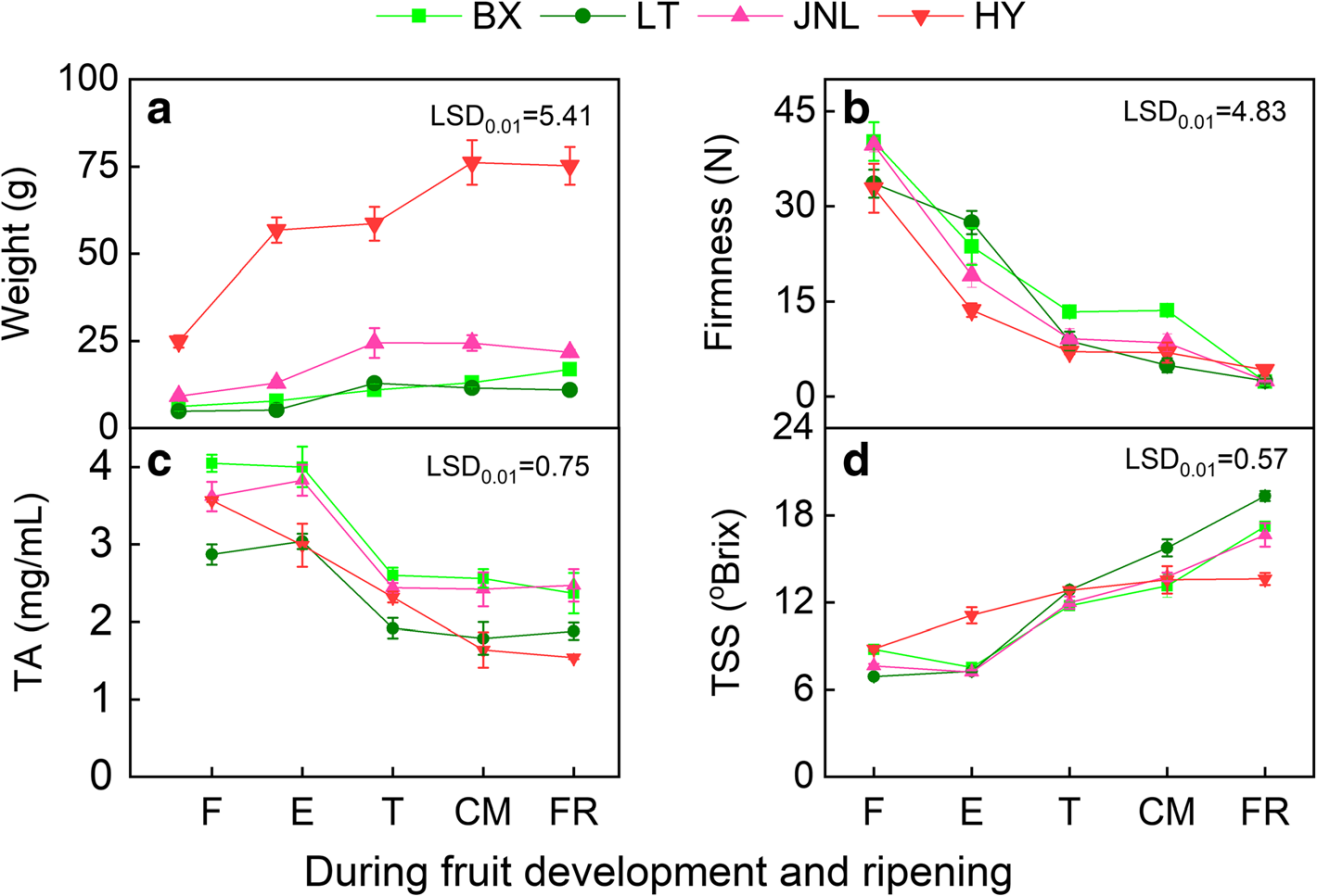
Changes in basic quality indexes during fruit development and ripening. **a** Fruit weight. **b** fruit firmness. **c** TSS, total soluble solid. **d** TA, titratable acid. Error bars are ± SE of the means of three biological replicates. The least significant difference (LSD) test at the 1% level was performed for all pairwise comparisons

**Fig. 2:**
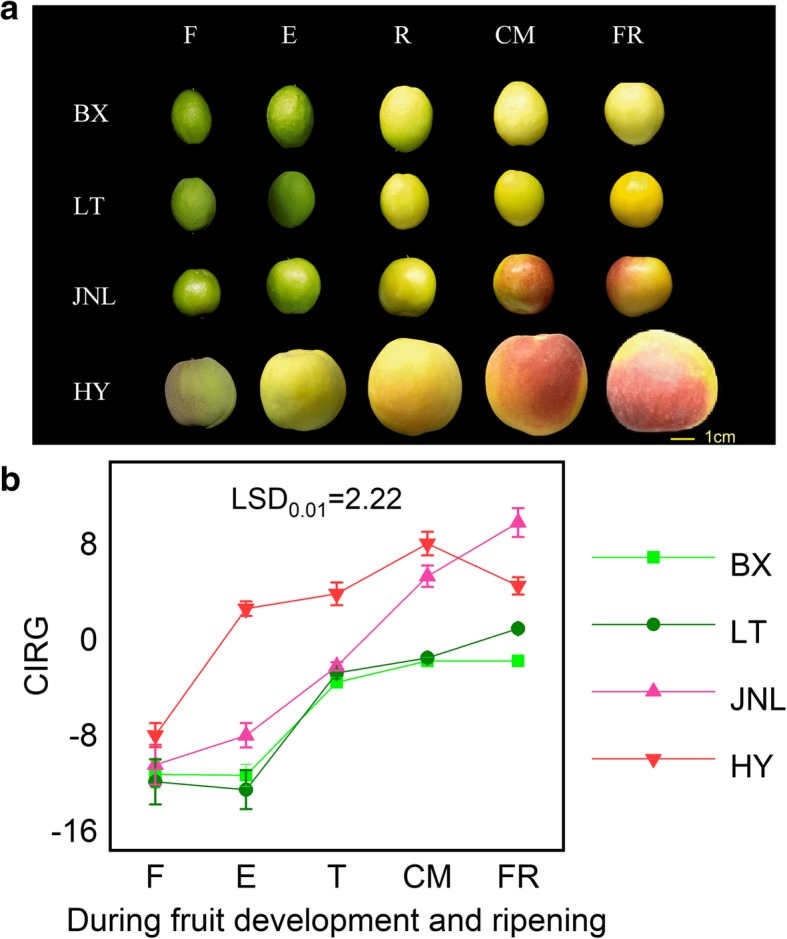
Changes in fruit color during development and ripening. **a** Color change of apricot fruit. **b** CIRG, color index for red grapes. Error bars are the ± SE of the means of three biological replicates. The least significant difference (LSD) test at the 1% level was performed for all pairwise comparisons

From the fruitlet (F) to fully ripe (FR) stages, the fruit weight of all experimental cultivars increased rapidly and presented a typical S-shaped curve (Fig. 1a), whereas the fruit firmness declined sharply throughout the development period (Fig. 1b). The TSS concentration in all fruits increased remarkably with fruit development (Fig. 1c) and the TA concentration decreased significantly (Fig. 1d), which showed that the five developmental stages completely covered the full process of fruit development and ripening. The four apricot cultivars showed a gradual color change from the turning (T) stage. The a* value increased during ripening and was positive at the enlargement (E), commercial maturation (CM) and FR stages in ‘HY’ and ‘JNL’, indicating that the fruit was turning red, whereas the value was negative during the whole ripening process in ‘LT’ and ‘BX’ (Additional file 1: Table S1). At the CM stage, a blush appeared on the fruit skin of ‘JNL’ and ‘HY’, whereas the skins of ‘LT’ and ‘BX’ did not display blush (Fig. 2a). Generally, the color index for red grape (CIRG) is used to evaluate the redness of fruit. The CIRG values of all fruit peels increased with development, although the CIRG values of the ‘HY’ and ‘JNL’ fruit peels were significantly higher than those of ‘BX’ and ‘LT’ (Fig. 2b).

### Changes in the anthocyanin content during fruit development and ripening

**Fig. 3:**
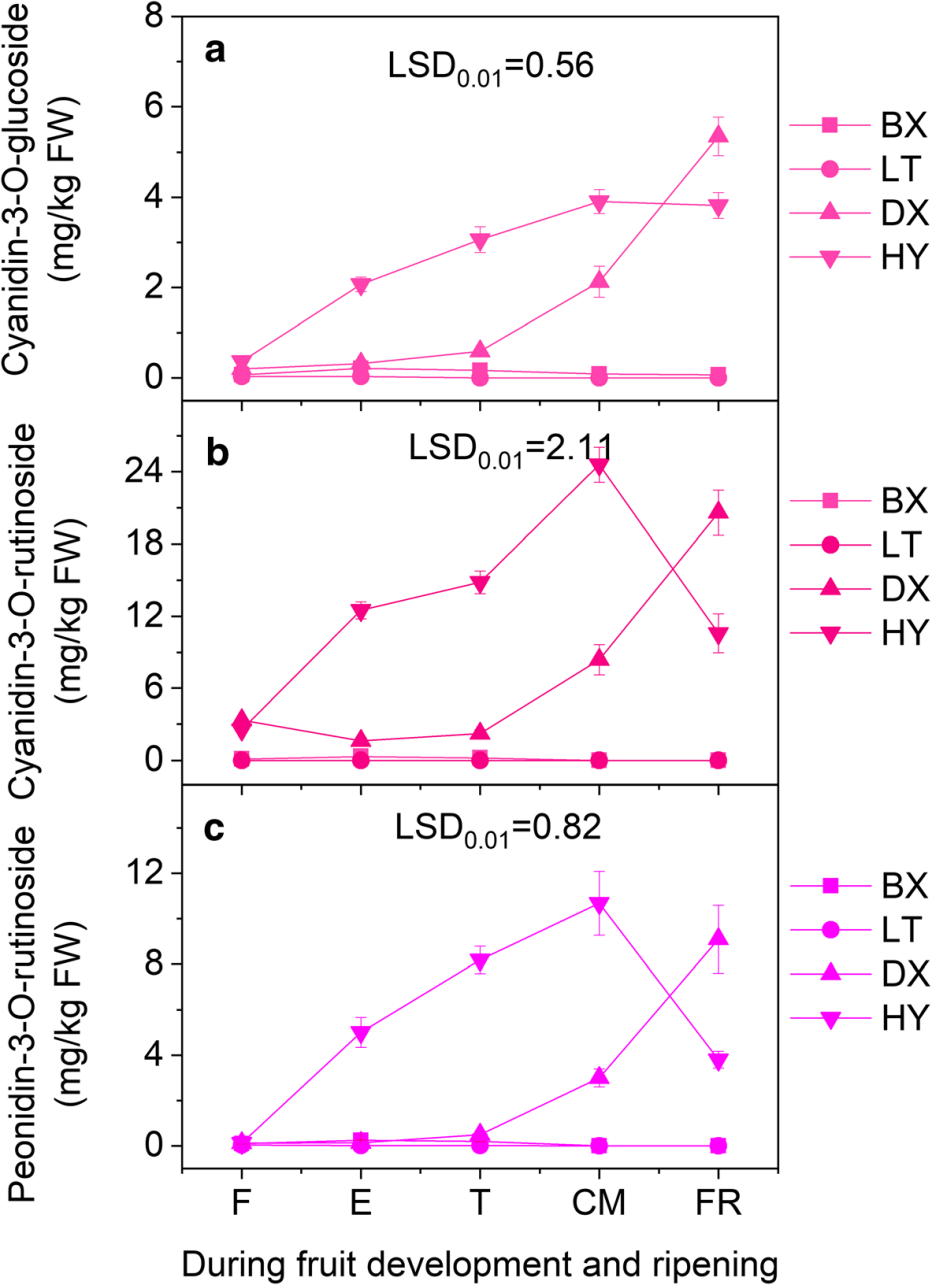
Changes of anthocyanidins in apricot fruit during development and ripening. Error bars are the ± SE of the means of three biological replicates. The least significant difference (LSD) test at the 1% level was performed for all pairwise comparisons

Three anthocyanins (cyanidin-3-O-glucoside, cyanidin-3-O-rutinoside and peonidin-3-O-rutinoside) were identified from the apricot peels (Fig. 3). During development and ripening, the anthocyanin contents of ‘BX’ and ‘LT’ remained very low, whereas those of ‘JNL’ and ‘HY’ increased significantly. For ripe fruit, the total anthocyanin concentrations in ‘JNL’ and ‘HY’ were 35.05 mg/kg·FW and 39.17 mg/kg·FW, respectively, which were significantly higher than those in ‘BX’ (0.09 mg/kg·FW) and ‘LT’ (0.07 mg/kg·FW). Interestingly, we detected cyanidin-3-O-rutinoside in the red cultivars; it accounted for 58.8% and 62.7% of the total anthocyanins in ‘JNL’ and ‘HY’, respectively, but was not detected in the ‘BX’ and ‘LT’ fruit peels.

### Screening of candidate genes by WGCNA

**Fig. 4:**
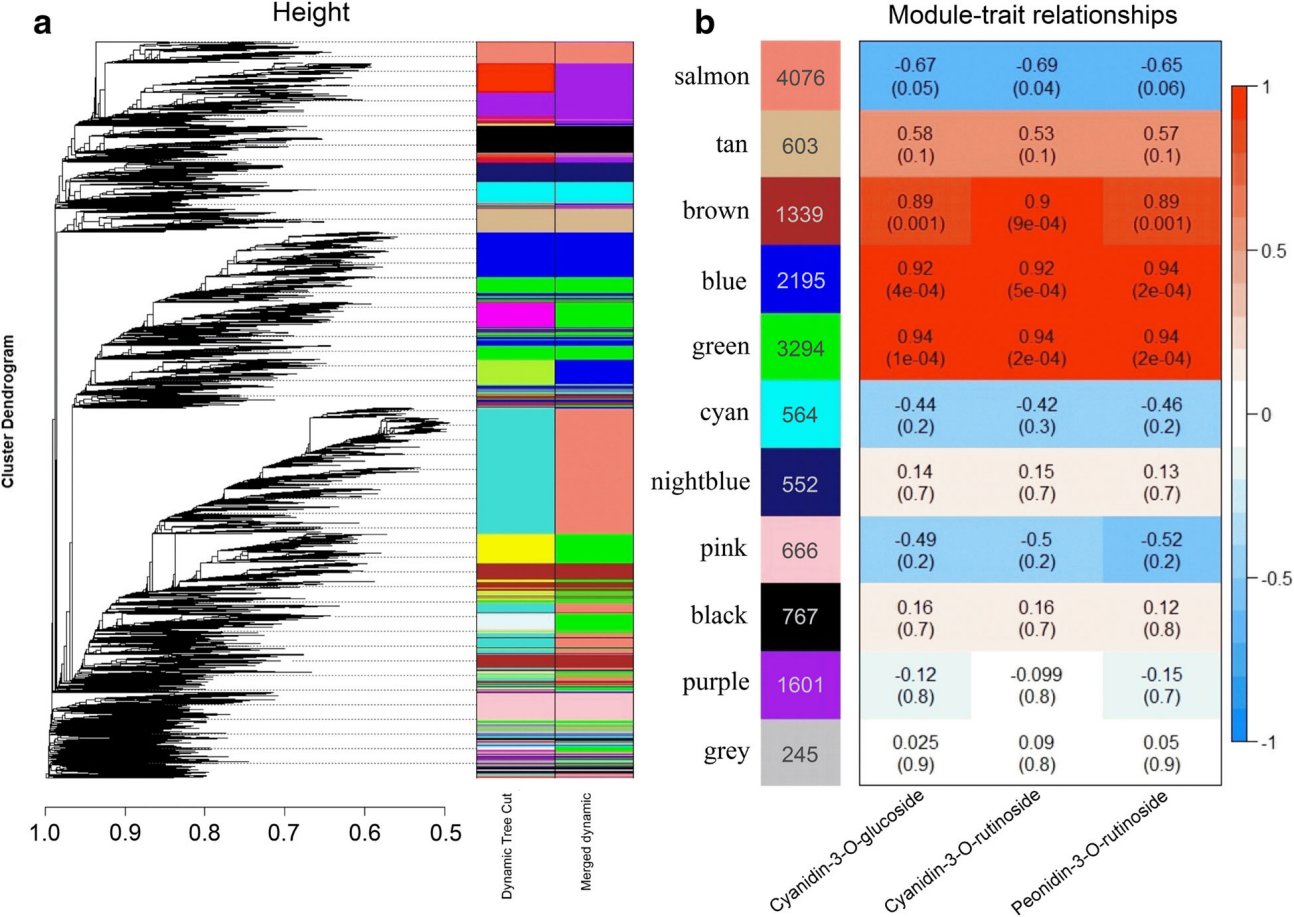
Weighted gene coexpression network analysis of apricot transcriptomes during fruit ripening. **a** Hierarchical cluster tree showing 11 coexpressed gene modules. Each leaf in the tree represents one gene. **b** Module-anthocyanin correlations and corresponding *p*-values. The left panel shows 11 modules and the numbers of their member genes. The right panel is a color scale for module trait correlation from − 1 to 1

**Fig. 5:**
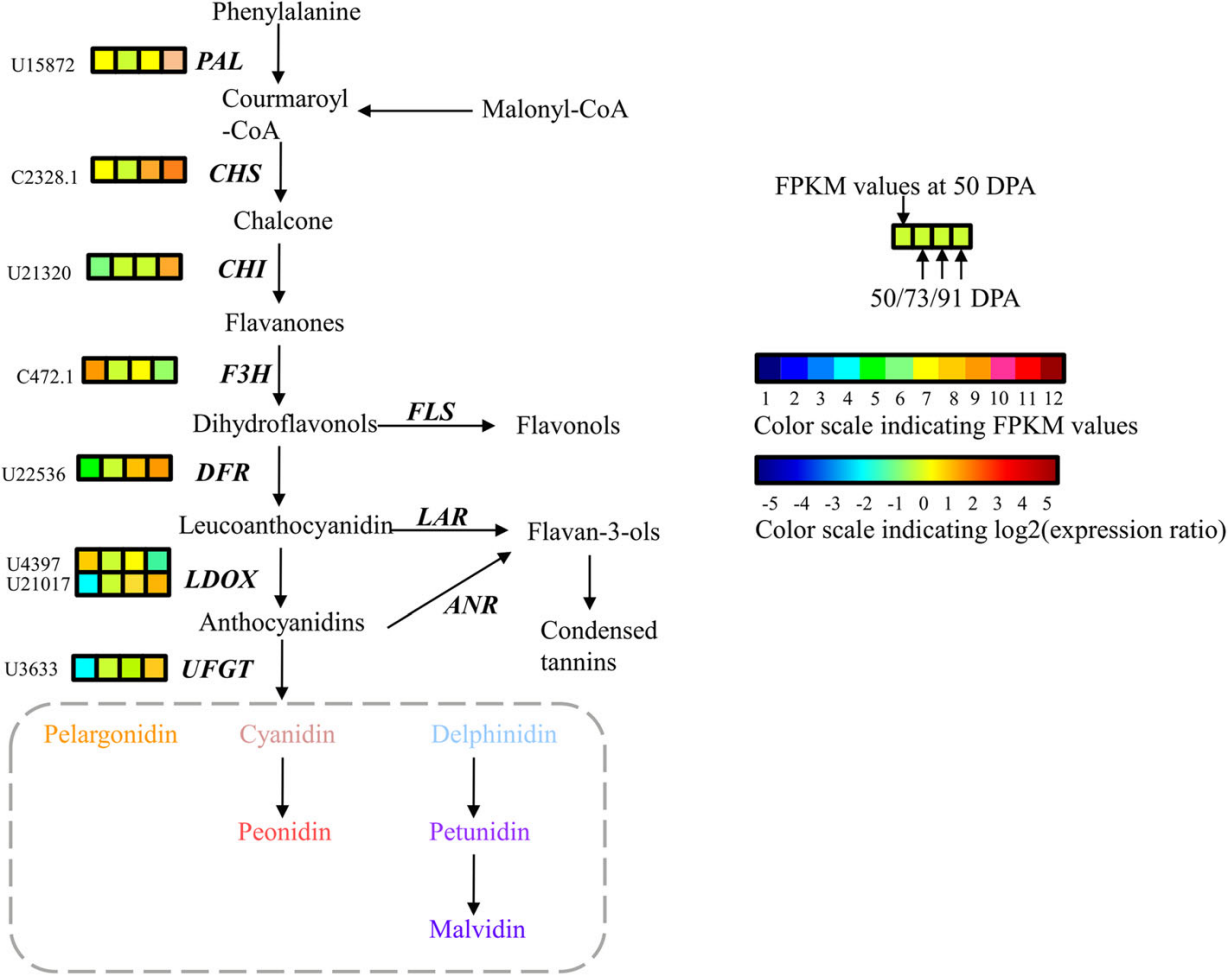
Anthocyanin metabolism scheme in apricot fruit during ripening. The expression patterns of each Unigene are shown as 4 grids, with the left grid representing the FPKM value at 50 DPA, and the second to fourth grids from the left to the right representing the relative log2 (expression ratio) at 50 DPA, 73 DPA, and 91 DPA, respectively. The grids with 12 different colors from blue to wine show the absolute expression magnitude at 50 DPA, with FPKM values of 0–1, 1–2, 2–4, 4–8, 8–16, 16–32, 32–64, 64–128, 128–256, 256–512, 512–1024 and over 1024 represented by colors 1 to 12, respectively

We performed a WGCNA to investigate the transcription factors and structural genes responsible for anthocyanin biosynthesis. We found that the blue and green modules were significantly positively correlated with cyanidin-3-O-glucoside, with correlation coefficients of 0.92 (e-value = 4 × 10^− 4^) and 0.94 (e-value = 1 × 10^− 4^), respectively. Similar significant correlations were found between the two modules and cyanidin-3-O-rutinoside and peonidin-3-O-rutinoside (Fig. 4). Based on the correlation coefficients between genes in the blue and green modules and the anthocyanin compounds, and changes in *Fragments Per Kilobase* of transcript per *Million* fragments mapped (FPKM) values of these genes during fruit ripening (Fig. 5), one U1373 gene encoding MYB was located in the blue module, and seven structural genes were identified as putative candidate genes for anthocyanin biosynthesis in apricots, including one chalcone isomerase (CHI: U21320) and one UDP-glucose: flavonoid 3-O-glucosyltransferase (UFGT: U3633) located in the blue module and one phenylalanine ammonia lyase (PAL: U15872), one chalcone synthase (CHS: CL2328.1), one flavonoid 3-hydroxylase (F3H: CL472.1), one dihydroflavonol 4-reductase (DFR: U22536), and one leucoanthocyanidin dioxygenase (LDOX: U21017) located in the green module (Additional file 2: Table S2).

### Phylogenetic and conserved motif analyses and subcellular localization of *PaMYB10*

**Fig. 6:**
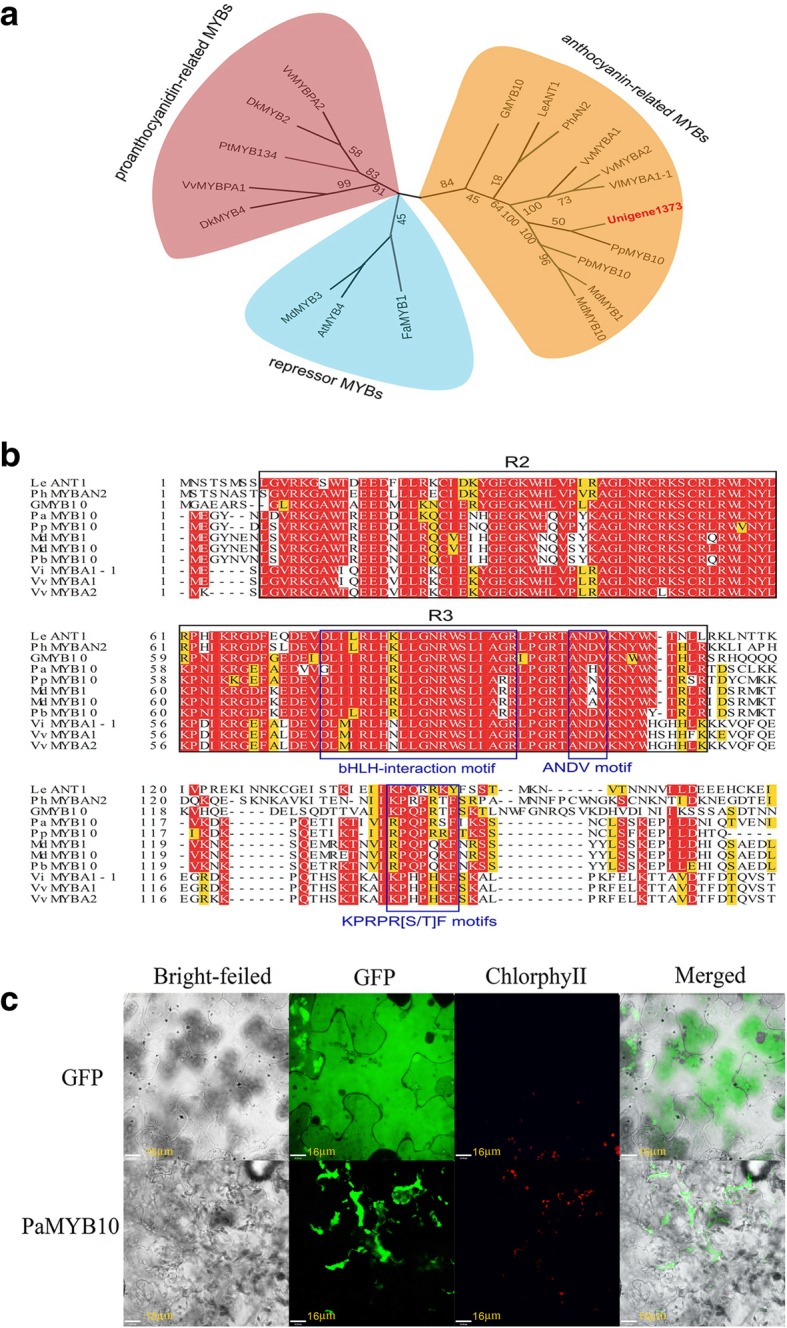
Sequence analysis and subcellular localization of *PaMYB10.* **a** Phylogenetic tree of the U1373 gene and other MYB transcription factors from different plants. **b** Amino acid sequence alignment of *PaMYB10* with other species. The red font indicates 100% homology, the black boxes indicate the R2R3 domain, and the blue boxes indicate the bHLH-interaction domain, ANDV, and KPRPR [S/T] F motifs. **c** Subcellular localization of *PaMYB10* in *Nicotiana benthamiana* leaves. The enhanced green fluorescent protein (GFP)-fused *PaMYB10* was transiently expressed in *N. benthamiana* leaves and imaged with a confocal fluorescence microscope

A bootstrapped circular phylogenetic tree was constructed based on 148 MYB genes from the apricot transcriptome and MYB transcription factors in other species. The tree showed that the 148 MYB TFs were distributed into five clades (Additional file 3: Figure S1), including flavonoid-related MYB, water stress-related MYB, phenol-related MYB, cell wall composition-related MYB, and some unknown function MYB clades. U1373 was clearly clustered with other MYB transcription factors involved in flavonoid synthesis. To further verify the results, another phylogenetic tree was generated based on U1373 and 18 flavonoid-related MYB transcription factors from other species. The second phylogenetic tree showed that U1373 belonged to the anthocyanin-related MYB clade and clearly clustered with PpMYB10, showing high homology with PbMYB10 in pear (*Pyrus bretschneideri*) fruit and MdMYB1 and MdMYB10 in apples (*Malus domestica*) (Fig. 6a) [27, 28]. Therefore, U1373 was designated *PaMYB10*.

Furthermore, the full-length U1373 was isolated from the peel of ‘JNL’ using RACE. The sequence was 675 bp in length and contained a complete open reading frame encoding 225 amino acids. Subsequently, comparison of the deduced amino acid sequence of *PaMYB10* with those of several anthocyanin-related MYB transcription factors from other plants revealed a high degree of sequence similarity in the R2 and R3 DNA-binding domains of these proteins. As shown in Fig. 6b, a conserved KPRPR [S/T] F motif was also present in the R2R3 domain of the PaMYB10 C-terminus. Further analysis showed that the R3 DNA-binding domain contained an R/B-like bHLH interaction motif ([DE]Lx2[RK]x3Lx6Lx3R) and an ANDV motif.

To determine the subcellular localization of *PaMYB10*, tobacco (*Nicotiana tabacum*) leaves bombarded with the recombinant vector pC1301-GFP-MYB10 and control vector pC1301-GFP were observed under a confocal laser scanning microscope. We found that the tobacco leaves with the control vector pC1301-GFP exhibited fluorescence throughout the entire cell, whereas those with the recombinant vector p163-GFP-MYB10 displayed strong fluorescence in the nucleus. These results demonstrated that the expression site of this gene was in the nucleus (Fig. 6c).

### Transcript levels of *PaMYB10* and structural genes related to anthocyanin synthesis

**Fig. 7:**
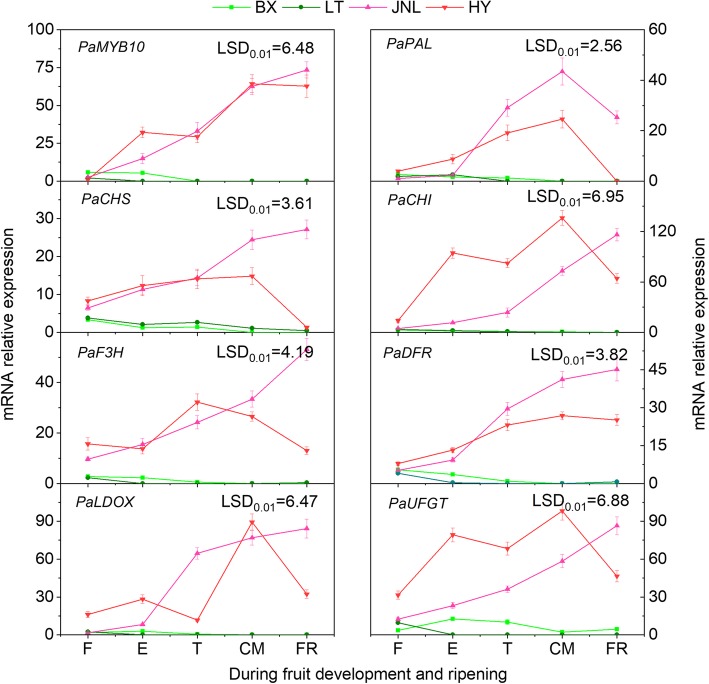
The transcript levels of genes involved in anthocyanin biosynthesis in fruit peels. Error bars are the ± SE of the means of three biological replicates. The least significant difference (LSD) test at the 1% level was performed for all pairwise comparisons

The results showed that the transcript levels of all genes in the peels of the blushed cultivars (‘JNL’ and ‘HY’) were significantly higher than those in the peels of the nonblushed cultivars (‘BX’ and ‘LT’) (*P* < 0.05) (Fig. 7). The transcript levels of *PaMYB10*, *PaPAL*, *PaCHS*, *PaCHI*, *PaF3H*, *PaDFR*, *PaLDOX* and *PaUFGT* were low in ‘BX’ and ‘LT’, with only slight upregulation observed in the F and T stages. However, the expression levels of *PaMYB10* and the seven structural genes in ‘JNL’ and ‘HY’ all increased remarkably during the whole developmental period, whereas the expression patterns of *PaMYB10* and most of the structural genes in ‘HY’ showed a double-peak pattern and reached their highest values in the CM stage.

### The effect of bagging on gene expression and anthocyanin accumulation in the ‘JNL’ fruit

**Fig. 8:**
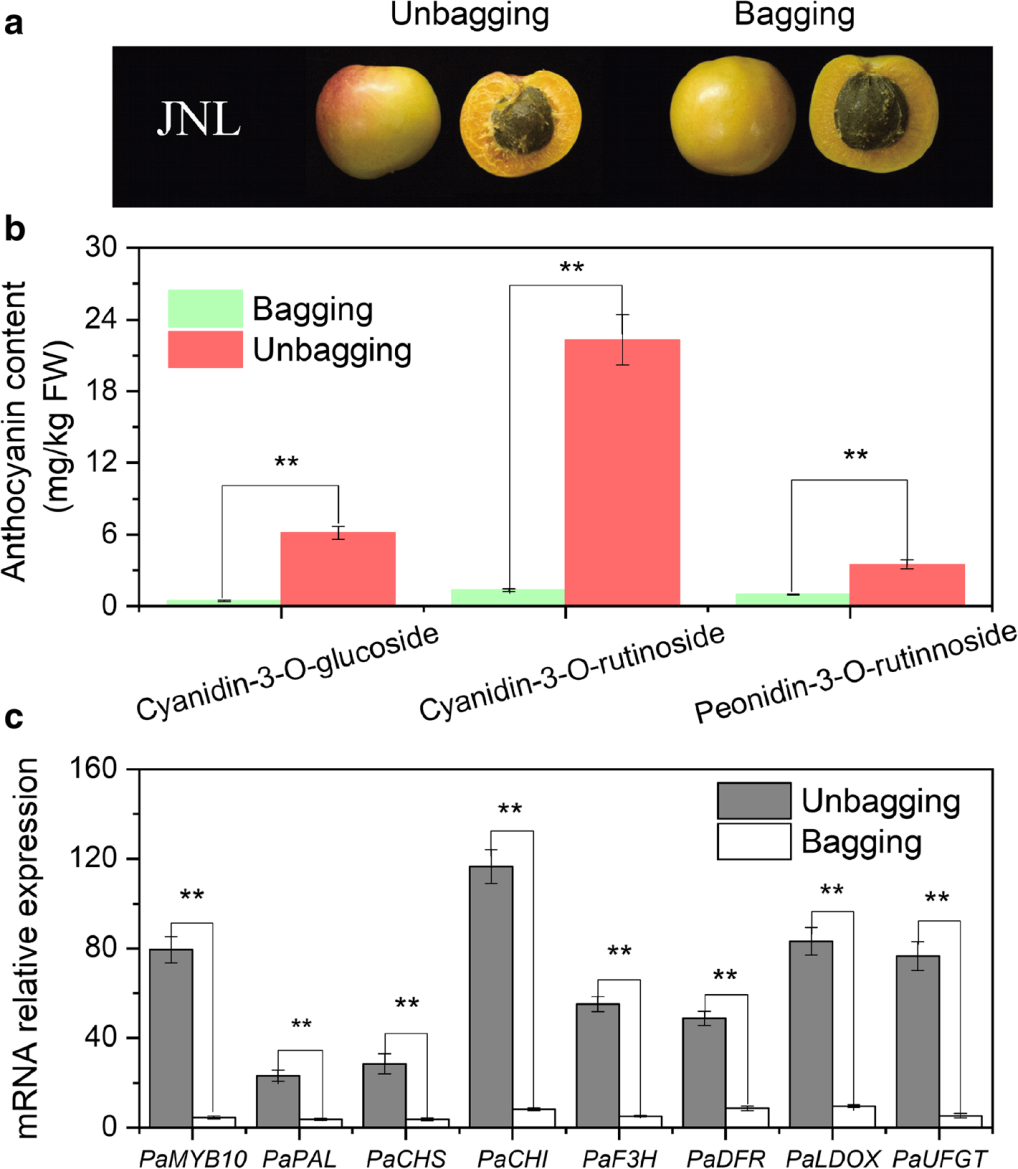
Effect of bagging on the anthocyanin content and the transcript levels of genes related to anthocyanin biosynthesis in ‘JNL’. **a** Fruit color. **b** Changes in the anthocyanin content. **c** Transcripts levels of genes related to anthocyanin biosynthesis. Error bars are the ± SE of the means of three biological replicates. The double asterisk indicates statistically significant differences by Duncan’s test at the 1% level

The a* value in bagged fruits (5.87) was significantly lower than that in nonbagged fruits (20.84) (Additional file 1: Table S1), and the CIRG values also decreased from 1.20 to 0.90. No blush was observed in the bagged ‘JNL’ fruit skin (Fig. 8a). The cyanidin-3-O-glucoside, cyanidin-3-O-rutinoside and peonidin-3-O-rutinoside contents in bagged fruits were extremely reduced from 35.05 mg/kg FW to 2.17 mg/kg FW in the ‘JNL’ peel (Fig. 8b), and the *PaMYB10*, *PaPAL*, *PaCHS*, *PaCHI*, *PaF3H*, *PaDFR*, *PaLDOX* and *PaUFGT* expression levels were also significantly lower than those in the nonbagged fruits (*P* < 0.05) (Fig. 8c).

### Transient *PaMYB10* overexpression increases anthocyanin production in the ‘LT’ fruit peel

**Fig. 9:**
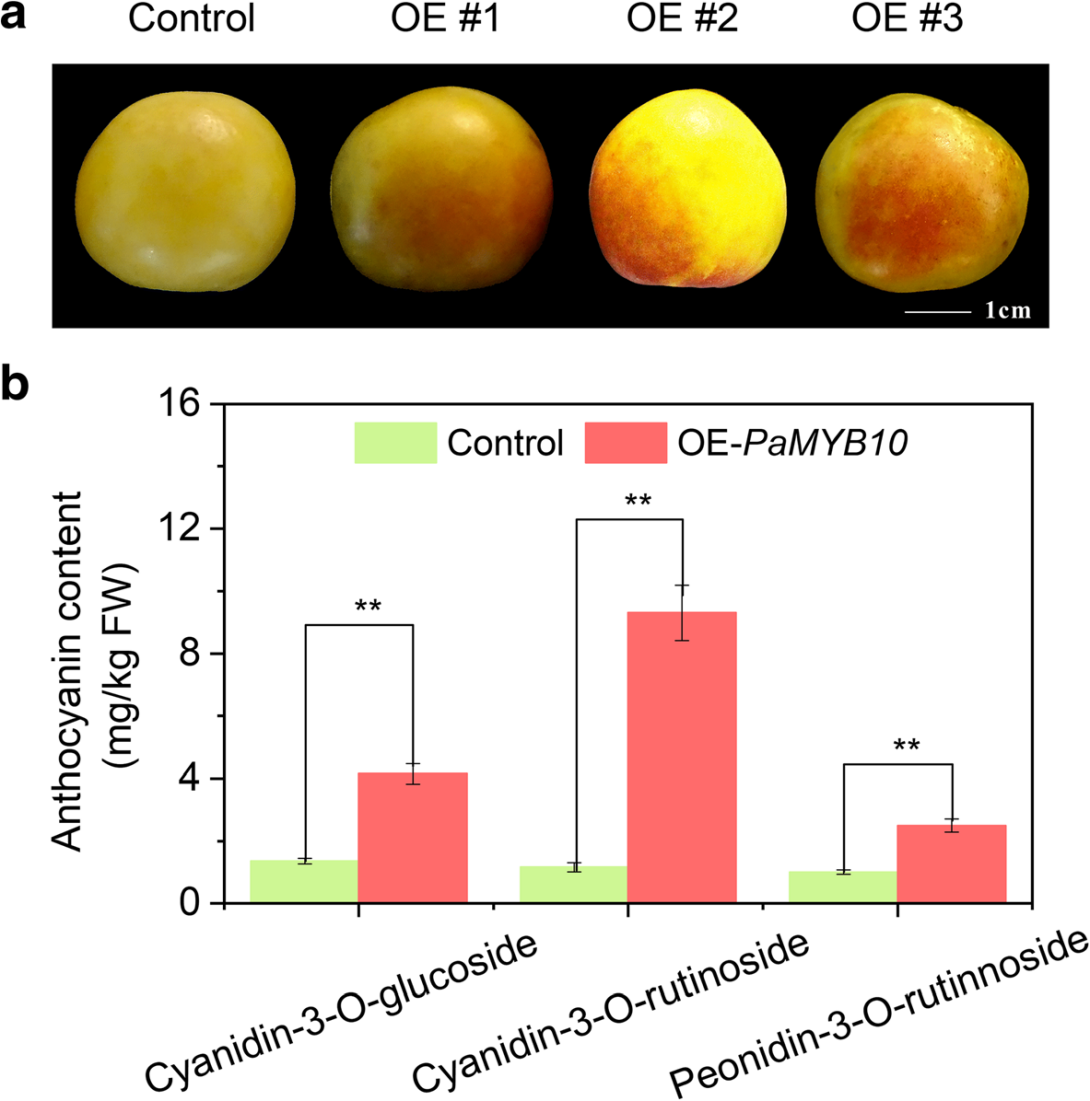
Transient overexpression in apricot fruit. **a** Fruits transformed by the empty vector (Control) and overexpression *PaMYB10* vector (OE#1, 2 and 3). **b** change in the anthocyanin content in fruit with transient overexpression and the control. Error bars are the ± SE of the means of three biological replicates. The double asterisk indicates statistically significant differences by Duncan’s test at the 1% level

*PaMYB10* was transiently overexpressed in the ‘LT’ fruit peel at the CM stage. The fruit peel presented with a red blush, whereas the control fruit skin did not present with a blush (Fig. 9a). Compared with those of the control, the cyanidin-3-O-glucoside, cyanidin-3-O-rutinoside and peonidin-3-O-rutinoside contents were significantly increased by 2.2-fold, 3-fold and 2.4-fold, respectively, in the *PaMYB10*-overexpressed fruits (Fig. 9b).

## Discussion

Apricots with a blush on orange or yellow skin are becoming more and more popular in the market due to their colorful appearance and excellent nutritional value [29]; however, the mechanism underlying the development of these cultivars has remained elusive. In this study, we compared the anthocyanin composition and content among apricot cultivars with and without a blush. Based on the WGCNA, gene expression and gene structural analyses and transgenic experiments, we identified the R2R3-MYB TF PaMYB10 as a master regulator that controlled the entire anthocyanin biosynthesis process during apricot fruit ripening.

Our chemical analysis demonstrated that the total anthocyanin content in apricots with red blushed skin as well as the individual cyanidin-3-O-glucoside, cyanidin-3-O-rutinoside and peonidin-3-O-rutinoside contents were significantly higher than those found in apricots without a blush (Fig. 3). In particular, remarkably high cyanidin-3-O-rutinoside levels were observed in red blushed cultivars during fruit ripening but were undetectable throughout the whole ripening process in cultivars without a blush. These results showed that high levels of the three anthocyanins determined the blush coloration of the apricot fruit skin and that cyanidin-3-O-rutinoside played the dominant role in blush formation. As shown in Fig. 2a, the ‘JNL’ and ‘HY’ fruit skins turned red after the T stage, which showed that the blush depended on fruit ripening. Thus, to understand the molecular mechanism of blush formation, the T, CM and FR stages of ‘JNL’ fruits, which represented the full ripening process, were used for transcriptome sequencing. Using WGCNA, we identified one MYB gene and seven structural genes related to anthocyanin biosynthesis in the blue and green modules that were significantly positively correlated with cyanidin-3-O-glucoside, cyanidin-3-O-rutinoside and peonidin-3-O-rutinoside (Fig. 4). MYB was considered the hub gene in the anthocyanin metabolic network, which suggested a direct connection between MYB and anthocyanin biosynthesis in apricot fruit.

For functional studies, full-length *PaMYB10* cDNA was cloned from ‘JNL’. In the subsequent phylogenetic analysis, *PaMYB10* was found to cluster with *PpMYB10, PbMYB10, MdMYB1* and *MdMYB10* (Fig. 6a), which are known to be positively associated with anthocyanin production [27, 28, 30, 31]. Sequence alignment found that the apricot *PaMYB10* contained the typical R2 and R3 structural domains similar to other MYB members in the same cluster (Fig. 6a). Sequence analysis also showed that *PaMYB10* contained an ANDV motif in the R3 domain, which is an identifier for anthocyanin-related MYBs [27]. In addition to the R2R3 domain, the KPRPR [S/T] F motif, which is a R2R3-MYB specific structure [22], was found in *PaMYB10*. Finally, the subcellular localization analysis revealed that *PaMYB10*-GFP was distributed in the nucleus (Fig. 6c). These results suggested that *PaMYB10* may play an important role in regulating anthocyanin synthesis in apricot fruit.

We also compared the expression levels of *PaMYB10* and structural genes related to anthocyanin biosynthesis between blushed and nonblushed apricots and found that the transcript levels of all genes in the apricots with blush were significantly higher than those in the apricots without blush during the fruit ripening process. These findings were consistent with the accumulation of anthocyanins, which proved that these genes were related to anthocyanin biosynthesis (Fig. 7). At the same time, we demonstrated that bagging resulted in a significant decrease in the accumulation of anthocyanins in ‘JNL’ and prevented blush formation in the fruit skin (Fig. 8b), which coincided with a dramatic decrease in the transcript levels of *PaMYB10* and the structural genes (Fig. 8c). These results further confirmed that *PaMYB10* was a key gene involved in the control of anthocyanin biosynthesis and simultaneously suggested that light could affect blush formation on apricots. These findings are in agreement with previous reports in other fruits linking the light-mediated mechanism involved in the regulation of anthocyanin biosynthesis with ripening-related anthocyanin accumulation [14].

In plants, transcriptional regulation of structural genes by transcription factors appears to be the major mechanism controlling anthocyanin biosynthesis [14, 32, 33]. Most MYBs involved in the control of anthocyanin biosynthesis are positive regulators that enhance the expression of structural anthocyanin pathway genes. However, repressors have also been characterized, such as *VvMYB4* in the berries of grapevines and *FaMYB1* in strawberries *(Fragaria x ananassa* Duch.) [23, 34]. In the present study, transient overexpression of a single *PaMYB10* gene in ‘LT’ apricots promoted blush formation and significantly increased the transcript abundance of *PaMYB10* and structural genes responsible for anthocyanin biosynthesis. These results suggested a central positive role for *PaMYB10* in regulation of anthocyanin synthesis.

R2R3 MYB TF regulation can occur at different steps of the anthocyanin biosynthesis pathway. For example, R2R3 MYB TFs in perilla (*Perilla frutescens*) control transcription of all structural genes involved in anthocyanin biosynthesis [35]. Overexpression of *AtPAP1* and *AtPAP2* strongly induce anthocyanin accumulation in *Arabidopsis* as a result of upregulation of all genes in the anthocyanin biosynthetic pathway [36, 37]. Similarly, our results showed that almost all genes responsible for anthocyanin biosynthesis were up-regulated in overexpressed apricot fruit of *PaMYB10*, which suggested the global regulation role of *PaMYB10* for the whole biosynthesis pathway of anthocyanin biosynthesis in apricot, but future work is still required to investigate which genes are targeted by the MYB TF.

In general, regulation of the anthocyanin biosynthesis pathway is controlled by the MYB-bHLH-WD40 (MBW) complex [7, 23]. But some anthocyanin biosynthesis-related MYB transcription factors that are activated without a bHLH partner have been identified in grapevines and *Arabidopsis thaliana* [24, 38]. In the present study, the amino acid signature ([DE]Lx2[RK]x3Lx6Lx3R) of the R3 domain, which was used to connect the MYB and bHLH proteins [21], was found in the PaMYB10 protein, suggesting a potential interaction between the bHLH protein and PaMYB (Fig. 6b), so more evidence should be provided for whether other TFs may be coordinately involved in the process.

## Conclusions

In the study, we found that the red blushed skin of apricots is attributed to the accumulations of cyanidin-3-O-glucoside, cyanidin-3-O-rutinoside and peonidin-3-O-rutinoside during fruit ripening. The R2R3 MYB TF *PaMYB10* was identified as a central gene related to the regulation of anthocyanin biosynthesis and blush formation, and the process is regulated by light.

## Methods

### Plant materials

Four varieties of apricot [‘Jianali’ (JNL) and ‘Hongyu’ (HY) (fruit with blushed skin) and ‘Baixing’ (BX) and ‘Luntaixiaobaixing’ (LT) (fruit without blushed skin)] cultivated at the National Fruit Tree Germplasm Repository, Academy of Xinjiang Agricultural Sciences, Luntai, Xingjiang, China, were used as the experimental materials. Every six individual trees were treated as one replication, and fruits were harvested at the fruitlet (F, 30 DAB), enlargement (E, 45 DAB for ‘BX’, ‘LT’, ‘JNL’ and ‘HY’), turning (T, 60 DAB for ‘BX’ and ‘LT’ and 54 DAB for ‘JNL’ and ‘HY’), commercial maturation (CM, 75 DAB for ‘BX’ and ‘LT’ and 72 DAB for ‘JNL’ and ‘HY’) and fully ripe (FR, 90 DAB for ‘BX’ and ‘LT’ and 82 DAB for ‘JNL’ and ‘HY’) stages. In addition, fifty ‘JNL’ fruits were bagged before the T stage and harvested at the FR stage. Twenty of the fifty harvested fruits were used to measure basic physiological indexes, such as the fruit size, chromatic aberration, total soluble solids (TSS), and titratable acidity (TA). Three biological replicates for each sample were used. The peel and pulp were separated from the remaining 30 sampled fruits, immediately frozen in liquid nitrogen, and stored at − 80 °C prior to use.

### Anthocyanin extraction and identification

Total anthocyanins were extracted from the fruit peels according to the methods described by our previous study with slight modifications [39]. Frozen peels (1 g) were ground to a fine powder by an IKA A11 analytical grinder (IKA, Staufen, Baden-Württemberg, Germany) and extracted using 3 mL of 2% formic acid for 24 h at 4 °C in the dark. The extraction was evaporated using a rotary evaporator for the next step. The leachate residue was dissolved in methanolic hydrochloric acid solution (pH = 2.3) and then adjusted to a final volume of 1.5 mL. Subsequently, the clear supernatant was collected and filtered to remove cell debris. Anthocyanins in the samples were analyzed using a Waters HPLC system with a diode array detector (Waters, Milford, MA, USA) at 40 °C. Each sample (20 μL) was loaded onto a reversed-phase C_18_ analytical column (4.6 mm i.d. × 250 mm i.d., 5 μm) (Nacalai Tesque, Kyoto, Japan) and eluted using a mobile phase consisting of solvent A (2% formic acid in water) and solvent B (2% formic acid and 98% acetonitrile) for 40 min at a flow rate of 1 mL per min. The effluent absorbance was monitored at 525 nm using the Genesys 10 UV spectrophotometer (Thermo Spectronic, Rochester, NY, USA). The concentration was calculated by the standard curve method and expressed as mg/kg·FW. Three biological replicates for each sample were used.

### RNA extraction and cDNA synthesis

Total peel RNA was extracted from the fruit using a total RNA kit (RNeasy Plant Mini Kit, Qiagen, Hilden, Germany). RNA samples with an OD260:OD280 between 1.80 and 2.0, an OD260:OD230 > 1.5 and no discernible degradation were used for cDNA reverse transcription. The RNA was treated with the TURBO DNA-free™ Kit (Ambion, Austin, TX, USA) following the manufacturer’s instructions. Subsequently, first-strand cDNA was synthesized from 1 μg of total RNA using the RevertAid First Strand cDNA Synthesis Kit (Invitrogen, Tokyo, Japan) according to the manufacturer’s protocol.

### Transcriptome sequencing and weighted gene coexpression network analysis (WGCNA)

‘JNL’ peel transcriptome sequencing was conducted on the Illumina HiSeq2000 (Illumina, USA) platform. Sequence assembly and annotation were performed according to our previous work [2]. The WGCNA (v1.29) package in R was employed to construct coexpression networks and test the association with the anthocyanins. Sequences with an average NRPKM > 1 from three replicates were used for the WGCNA. The modules were obtained using the automatic network construction function blockwise with default settings, except that the soft power was 16, the min module size was 30, and the merge cut height was 0.25.

### Phylogenetic analysis

A total of 148 MYB sequence fragments were mined from our transcriptome data, and a phylogenetic tree was constructed with MYB transcription factors from other species. After homology alignment, another unrooted phylogenetic tree was constructed using the neighbor-joining (NJ) criteria and verified using the maximum likelihood (ML) method. A total of 1000 bootstrap replicates was performed based on multiple alignments of amino acid (AA) sequence U1373 from JNL and 18 flavonoid-related MYB transcription factors from other higher plants (Additional file 4: Table S3). Multiple protein sequence alignments were performed using ClustalW, and the phylogenetic trees were constructed using MEGA 7.0.

### Quantitative real-time PCR analysis

Eight pairs of primers (Additional file 5 Table S5) were designed using Primer Premier 5.0, and qRT-PCR was performed using the Light Cycler System (Roche LightCycler® 480; Roche Diagnostics). All reactions were performed using the LightCycler® 480 SYBR Green I Master Mix (Roche Diagnostics) according to the procedure described by the manufacturer. The qRT-PCR program was 95 °C for 30 s, followed by 40 cycles at 95 °C for 5 s and 60 °C for 30 s. The reaction volume was 25 μL and contained 12.5 μL of SYBR Premix Ex Taq (TaKaRa, Dalian, China), 8.5 μL of deionized water, 1 μL of each primer, and 2 μL of diluted cDNA. Three biological replicates for each sample were used. Each sample was normalized using ribosomal RNA and actin as the internal control genes [39, 40]. The relative expression levels of the eight genes were calculated using the 2^-△△Ct^ method.

### Cloning of the full-length *PaMYB10* gene

The *PaMYB10* gene was isolated from the cDNA of ‘JNL’ using a RACE kit (Takara, Dalian, China) (Additional file 6 Table S5). The full-length *PaMYB10* was obtained by assembling the 3′ RACE and 5′ RACE sequences using the BigDye Terminator Version 3.1 Cycle Sequencing Kit (Applied Biosystems) with the ABI PRISM 3100 Genetic Analyzer (Applied Biosystems). The amplification product was cloned into the pMD18-T vector and then transformed into *Escherichia coli* DH5α (BioTeke, China). Afterwards, positive colonies were sequenced by Sangon Biological Engineering Company (Shanghai, China).

### Subcellular localization

The *PaMYB10* open reading frame (ORF) without the terminator was amplified with special primers (forward: 5′-GAGAACACGGGGGACTGGTACCCGGGGATCCA TGGAGGGCTATAACTTGGA-3′ and reverse: 5′-ACAGCTCCTCGCCCTTGCTC ACCATGTCGACCCTAGCTTCTTCTGAAACAT-3′) and ligated into the pC1301-GFP vector to generate the CAMV 35S:*PaMYB10*-GFP fusion construct. The recombinant vector (*PaMYB10*-GFP) and control vector (pC1301-GFP) were both transformed into tobacco by particle bombardment. After 16 to 18 h, fluorescence was detected with a confocal laser scanning microscope (Zeiss LSM 510 META, Jana, Germany).

### Overexpression vector construct and transient transformation in apricot fruit

The overexpression pMDC32 binary vector49 was constructed as previously described [41, 42]. The *PaMYB10* coding sequence was PCR amplified from ‘JNL’ cDNA using the primers 5′-ATGGAGGGTTATTTCGGTGTGAG-3′ (forward) and 5′-TACGTAGGAGATGTTGACTAGATCATTGC-3′ (reverse) and cloned using the pCR™8/GW/TOPO TA Cloning Kit (Invitrogen). After sequencing confirmation, the CDS was recombined into the pMDC32 binary vector49 using the Gateway LR Clonase II Enzyme mix (Invitrogen) to produce overexpression vectors. The vectors were transformed into *Agrobacterium tumefaciens* GV3101 and then resuspended at a final O.D. of 0.8 in liquid MS medium. One milliliter of the suspension was evenly injected into the ‘LT’ fruit at commercial mature stage. Thirty fruits were injected as the repeat, and fruits were injected with *Agrobacterium* carrying an empty vector as a control treatment. At 7–12 days after injection, the fruit peel surrounding the injection sites was collected and immediately frozen in liquid nitrogen and stored at 80 °C for determining the anthocyanin according to the method of above mentioned.

### Statistical analyses

Three biological replicates were performed for all experiments. The least significant difference (LSD) or Duncan’s test at the 1% level was analyzed by DPS (version 2.00; Zhejiang University, Hangzhou, China). Data are expressed as the mean of three biological replicates ± the standard deviation (SD).

## Additional files

Additional file 1: **Table S1.** Color indexes of apricot fruits at different developmental stages. (PDF 251 kb)

Additional file 2: **Table S2.** Candidate genes involved in anthocyanin biosynthesis in apricot fruit. (PDF 214 kb)

Additional file 3: **Figure S1.** Phylogenetic tree of gene sequences for 148 MYB genes from the apricot transcriptome and MYB transcription factors from other species. (PDF 823 kb)

Additional file 4: **Table S3.** Flavonoid-related MYB transcription factors using in phylogenetic analysis. (PDF 227 kb)

Additional file 5: **Table S4.** The primer sequences for qRT-PCR. (PDF 363 kb)

Additional file 6: **Table S5.** The primer sequences for 5′ RACE and 3′ RACE. (PDF 351 kb)

## Data Availability

The data sets supporting the results of this article are included within the article and its additional files. All sequences obtained from apricots were deposited to the NCBI Sequence Read Archive (SRA) repository under accession number PRJNA506502 and were released on November 27, 2018.

## Abbreviations

ANR: Anthocyanidin reductase
BX: Baixing
CHI: Chalcone isomerase
CHS: Chalcone synthase
DFR: Dihydroflavonol-4-reductase
F3H: Flavanone 3-hydroxylase
FLS: Llavonol synthase
HY: Hongyu
JNL: Jianali
LAR: Leucoanthocyanidin reductase
LDOX: Leucoanthocyanidin dioxygenase
LSD: Least significant difference
LT: Luntaixiaobaixing
PAL: Phenylalanine ammonia-lyase
TF: Transcription factors
UFGT: UDP-flavonoid glucosyl transferase
