# Supplementary material for: The R2R3-MYB transcription factor PaMYB10 is involved in anthocyanin biosynthesis in apricots and determines red blushed skin
Source: BMC Plant Biol. 2019 Jul 1;19:287. doi: 10.1186/s12870-019-1898-4 (PMC6604168; doi:10.1186/s12870-019-1898-4)
Supplement: Supplementary file 1 — Table S1. Color indexes of apricot fruits at different developmental stages. (PDF 251 kb) [file 12870_2019_1898_MOESM1_ESM.pdf]

Additional file 1: Table S1 Color index for red grapes of apricot fruit at different developmental stages.

Cultivars	Period	L*	a*	b*	c*	h
Baixing	F	48.68±1.98	-20.09±0.50	37.06±2.01	42.16±1.95	118.50±0.97
	E	48.85±1.93	-19.17±1.08	34.98±3.43	39.89±3.51	118.80±1.13
	T	68.64±0.82	-9.31±2.54	39.33±1.42	40.49±1.49	103.28±3.59
	CM	71.59±0.88	-4.48±2.17	38.26±3.11	38.58±2.94	96.84±3.52
	FR	72.98±2.00a	-4.13±1.23b	34.00±2.93b	34.26±3.01b	96.86±1.79b
Luntaixiaobai	F	46.80±1.50	-20.15±0.71	36.59±2.25	41.77±2.29	118.87±0.80
	E	45.69±1.66	-19.78±0.61	34.79±2.07	40.03±2.07	119.65±0.88
	T	64.21±2.20	-7.67±1.58	44.79±1.90	45.48±1.61	99.78±2.37
	CM	64.48±2.01	-4.26±3.91	47.66±2.80	47.99±2.62	95.26±4.76
	FR	62.98±1.99	3.33±2.18	50.54±3.59	50.68±3.65	86.26±2.33
Jianali	F	52.04±2.26	-19.01±1.73	35.3±3.35	40.09±3.74	118.31±0.75
	E	59.42±3.01	-18.22±1.09	38.93±2.32	43.03±2.46	115.10±0.95
	T	60.91±2.80	-4.68±3.50	36.36±2.44	38.47±5.68	97.19±5.70
	CM	58.37±10.90	11.91±14.76	37.53±6.90	40.80±2.56	73.92±18.86
	FR-Unbagging	56.64±8.33b	20.84±8.90a	37.08±11.56b	44.38±4.47a	59.07±18.90b
	FR-Bagging	65.03±3.29a	5.87±1.96b	43.36±2.05a	43.78±2.25b	82.37±2.26a
Hongyu	F	52.13±3.18	-13.38±5.96	32.62±5.31	35.45±7.00	111.15±6.85
	E	69.19±1.61	8.04±3.84	42.74±2.86	43.66±2.20	100.88±5.56
	T	66.14±4.40	11.68±4.34	44.55±4.60	46.28±3.97	75.06±6.04
	CM	62.05±9.38	21.69±8.55	42.81±10.35	49.41±3.82	62.07±15.70
	FR	65.11±0.99	12.78±0.29	42.51±1.44	45.14±1.29	72.57±0.40

[a] Data are expressed as means ± standard deviation of three biological replicates.

[b] Different letters between unbagging and bagging fruits for the same index present significant differences between them ($P < 0.01$).
