# Supplementary material for: The R2R3-MYB transcription factor PaMYB10 is involved in anthocyanin biosynthesis in apricots and determines red blushed skin
Source: BMC Plant Biol. 2019 Jul 1;19:287. doi: 10.1186/s12870-019-1898-4 (PMC6604168; doi:10.1186/s12870-019-1898-4)
Supplement: Supplementary file 2 — Table S2. Candidate genes involved in anthocyanin biosynthesis in apricot fruit. (PDF 214 kb) [file 12870_2019_1898_MOESM2_ESM.pdf]

Additional file 2: Table S2 Candidate genes involved in anthocyanin biosynthesis in apricot fruit.

Pathway	Gene	Gene ID	Enzyme	KO id (EC-No.)	Expression pattern
	PAL	U15872	Phenylalanine ammonia-lyase	K10775	up-down
	CHS	CL2328.1	Chalcone synthase	K00660	up
	CHI	U21320	Chalcone isomerase	--	up
Anthocyanin biosynthesis	DFR	CL472.1	Dihydroflavonol reductase	K00475	up
	F3H	U22536	Flavanone 3-hydroxylase	K13082	up-down
	LDOX	U21017	Leucoanthocyanidin dioxygenase	K05277	up-down
	UFGT	U3633	Uridine diphosphate-glucose:flavonoid 3-O-glucosyltransferase	K12930	up
