# Supplementary material for: The R2R3-MYB transcription factor PaMYB10 is involved in anthocyanin biosynthesis in apricots and determines red blushed skin
Source: BMC Plant Biol. 2019 Jul 1;19:287. doi: 10.1186/s12870-019-1898-4 (PMC6604168; doi:10.1186/s12870-019-1898-4)
Supplement: Supplementary file 4 — Table S3. Flavonoid-related MYB transcription factors using in phylogenetic analysis. (PDF 227 kb) [file 12870_2019_1898_MOESM4_ESM.pdf]

Additional file 4: Table S3 Flavonoid-related MYB transcription factors using in phylogenetic analysis.

Species	Current name	GenBank number	Protein (aa)
Prunus persica	PpMYB10	EU155160	224
Pyrus x bretschneideri	PbMYB10	KT601121	244
Malus domestica	MdMYB1	DQ886414	243
Malus domestica	MdMYB10	DQ267897	243
Malus domestica	MdMYB3	JN544704	310
Vitis vinifera	VvMYBA1	AB097924	250
Vitis vinifera	VvMYBA2	AB242302	265
Vitis vinifera	VvMYBPA1	AM259485	286
Vitis vinifera	VvMYBPA2	EU919682	284
Lycopersicon esculentum	LeANT1	AY348870	274
Petunia x hybrida	PhAn2	AF146702	255
Fragaria × ananassa	FaMYB1	AF401220	187
Gerbera hybrid	GMYB10	AJ554700	250
Vitis labrusca x Vitis vinifera	VlMYBA1-1	AB073010	250
Diospyros kaki	DkMyb2	AB503699	259
Diospyros kaki	DkMyb4	AB503701	277
Populus tremuloides	PtMYB134	FJ573151	289
Arabidopsis thaliana	AtMYB4	AF062860	282
