# Supplementary material for: The R2R3-MYB transcription factor PaMYB10 is involved in anthocyanin biosynthesis in apricots and determines red blushed skin
Source: BMC Plant Biol. 2019 Jul 1;19:287. doi: 10.1186/s12870-019-1898-4 (PMC6604168; doi:10.1186/s12870-019-1898-4)
Supplement: Supplementary file 5 — Table S4. The primer sequences for qRT-PCR. (PDF 363 kb) [file 12870_2019_1898_MOESM5_ESM.pdf]

Additional file 5: Table S4 The primer sequences for qRT-PCR.

Primer	Forward primer（5′ to 3′）	Reverse primer（5′ to 3′）
26 S	GATAACGCAGGTGTCCTAAGATGA	ATTCCGAAGGTCTAAAGGATCGA
ACT	GTTATTCTTCATCGtCGTCTTCG	CTTCACCATTCCAGTTCCATTGTC
PaMYB10	TTTGGAATCATTAAAGGGAG	GGAAATAGAAATTGTAGGGAC
PaPAL	CAGAGCAGCACAACCAAGACG	CTCCAAATGCCTCAAATCAATG
PaCHS	AACAAGGGTGCTCGTGTTCTC	GCTGCACCATCACCGAATAAG
PaCHI	TTGGCGTGTACTTGGAGGAT	TTCTCAAATGGACCTGTAACGA
PaF3H	GGGTGGTTTCATCGTCTC	GGTAATTGTGCCTGGGTC
PaDFR	TCTCACAGAGGAAGCAGC	GAGGAGGCAGAAAGCCAT
PaLDOX	TGCTGATTACATTGAGGCTAC	TGAGGGCAAACTGGGTAG
PaUFGT2	GCATCTTGACCAGCCTAT	CTTCCACCACCACATCTC
