# Supplementary material for: The R2R3-MYB transcription factor PaMYB10 is involved in anthocyanin biosynthesis in apricots and determines red blushed skin
Source: BMC Plant Biol. 2019 Jul 1;19:287. doi: 10.1186/s12870-019-1898-4 (PMC6604168; doi:10.1186/s12870-019-1898-4)
Supplement: Supplementary file 6 — Table S5. The primer sequences for 5′ RACE and 3′ RACE. (PDF 351 kb) [file 12870_2019_1898_MOESM6_ESM.pdf]

Additional file 6: Table S5 The primer sequences of 5’RACE and 3’RACE.

Primer	Sequence（5′ to 3′）
5’RACE B1373-1(GSP1)	TGATTCGCTGTCCTTC
5’RACE B1373-2(GSP2)	AGCCTTCCAGCAATCAAT
5’RACE B1373-3(GSP3)	TCCTAAAAGCCTGTGAAGC
3’RACE C090-1	GGCTTCACAGGCTTTTAGGAAACAGG
3’RACE C090-2	AGCGAATCATGTGAAAAATTATTGGAAC
