# Supplementary material for: The R2R3-MYB transcription factor PaMYB10 is involved in anthocyanin biosynthesis in apricots and determines red blushed skin
Source: BMC Plant Biol. 2019 Jul 1;19:287. doi: 10.1186/s12870-019-1898-4 (PMC6604168; doi:10.1186/s12870-019-1898-4)

Additional file 3: Fig. S1 Phylogenetic tree of 148 MYB genes from the apricot transcriptome and MYB transcription factors from other species. *Pyrus bretschneider*i (Pb); *Malus domestic*a (Md); *Prunus persica (*Pp); *Arabidopsis thaliana (*At); *Glycine max* (Linn.) Merr. (Gm)

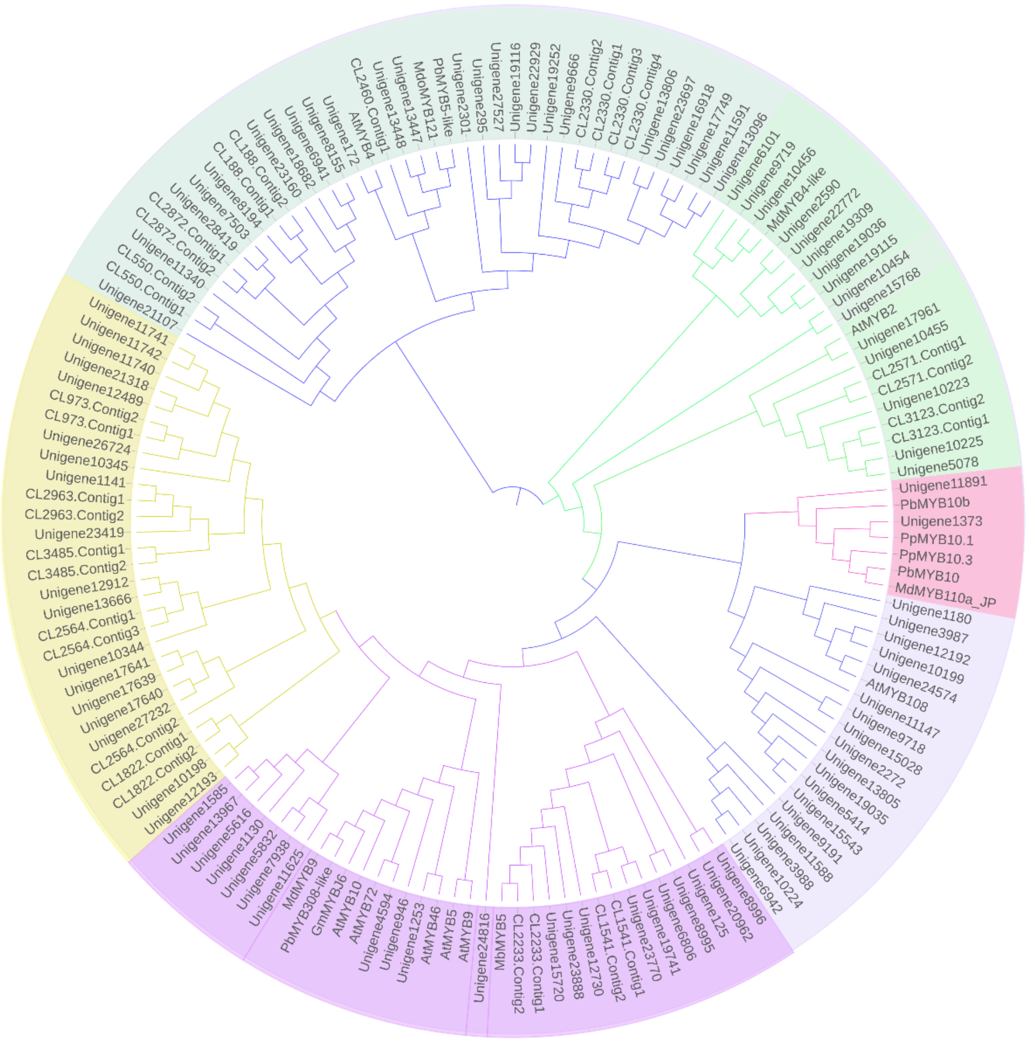

Supplement: Supplementary file 3 — Figure S1. Phylogenetic tree of gene sequences for 148 MYB genes from the apricot transcriptome and MYB transcription factors from other species. (PDF 823 kb) [file 12870_2019_1898_MOESM3_ESM.pdf]
